# Criteria of ultrasound-guided closed reduction with percutaneous pinning in unstable humeral lateral condylar fractures: a three-center retrospective cohort study

**DOI:** 10.3389/fped.2024.1373913

**Published:** 2024-03-06

**Authors:** Xiuming Huang, Chaoyu Liu, Guoqiang Jia, Jie Yao, Jianbing Xu

**Affiliations:** ^1^ Department of Orthopaedics, Ganzhou Maternal and Children’s Health Care Hospital, Ganzhou, China; ^2^ Department of Orthopaedics, Fuyang People’s Hospital of Anhui Medical University, Fuyang, China; ^3^ Department of Orthopaedics, The First Affiliated Hospital of the University of Science and Technology of China, Hefei, China

**Keywords:** ultrasound, humeri, closed reduction, lateral condylar fracture, pediatric

## Abstract

**Background:**

Interventions using ultrasound-guided closed reduction and percutaneous pinning (UG-CRPP) of humeral lateral condylar fractures (HLCFs) have been increasingly applied; however, their effectiveness for unstable HLCFs and the criteria for ultrasound outcomes remain unclear. This study assessed the outcomes of UG-CRPP for HLCFs and evaluated the success criteria in children.

**Methods:**

Data were retrospectively collected from 106 patients with unstable HLCFs admitted to three hospitals between January 2021 and August 2022. Fifty-five cases were left-sided and 51 cases were right-sided: 74 male patients and 32 female patients were included. Perioperative data, elbow function, complications, and criteria for UG-CRPP were analyzed.

**Results:**

The mean rate of UG-CRPP was 88%. The mean surgical time was 54.56 ± 21.07 min, and the mean fluoroscopy frequency was 9.25 ± 2.93 times. At the last follow-up, there were significant differences in elbow flexion between the affected side (135.82° ± 6.92°) and the unaffected side (140.58° ± 5.85°) (*p* = 0.01). The Mayo score of the affected side was 90.28° ± 4.97°, the Baumann angle was 71.4° ± 5.4°, condylar shaft angle was 39.9° ± 6.4°, and the carrying angle was 8.4° ± 3.6°. Seventy patients presented mild lateral spurs and 16 patients exhibited moderate spurs. Fourteen patients presented with pin infection, and one patient exhibited postoperative re-displacement. There was no premature physeal closure, varus, or valgus elbow deformity, delayed union, or non-union. Successful ultrasound-based outcome criteria for UG-CRPP were defined as follows: (i) absent or less than a cartilage thickness step on the cartilage hinge on coronal plane parallel articular surface scanning, (ii) no lateral displacement and intact distal end of the condylar and capitellum on coronal plane vertical articular surface scanning, (iii) no anteroposterior displacement and absent or less than a cartilage thickness step on sagittal plane vertical articular surface scanning, and (iv) intact posterior fracture line or less than a cortex step on posterolateral sagittal plane vertical articular surface scanning.

**Conclusion:**

UG-CRPP is a procedure with minimal blood loss, less invasive, cosmetic, and no radiation exposure. It yielded good outcomes in unstable HLCFs. The successful criteria make it suitable for clinical application.

## Background

Humeral lateral condylar fracture (HLCF) is the second most common type of elbow fracture in children (1). In 2008, Song et al. proposed a new classification for HLCF to guide clinical treatment according to fracture type: types 1–2 were recommended for conservative treatment, and types 3–5 were recommended for closed or open reduction with k-wires fixation (2). Arthrography has often been used previously to assist in the closed reduction of HLCFs (3–8). However, not only it is difficult to assess gaps and steps in the sagittal plane, but it is also challenging to dynamically evaluate the reduction process.

In recent years, various studies on ultrasound-guided closed reduction and percutaneous pinning (UG-CRPP) of elbow fractures in children have been reported (9–12). Ultrasonography has advantages of being radiation-free and allowing clear visualization of the distal cartilage and dynamic monitoring of the reduction process. It effectively evaluates alignment after reduction and has been applied at different centers for various types of displaced HLCFs (13–18). However, there has been no multicenter evaluation of the efficacy outcomes of UG-CRPP applied to unstable HLCFs, and the criteria for successful outcomes remain unclear. Therefore, this multicenter study summarizes the outcomes of UG-CRPP for unstable HLCFs and explores the success criteria for ultrasound guidance.

## Methods

### Clinical data

Cases of unstable HLCFs treated between January 2021 and August 2022 were retrospectively reviewed. The inclusion criteria were unstable type (types 3–5) according to the Song classification on x-ray, age < 14 years old, signed informed consent, and a follow-up period longer than 6 months. Exclusion criteria included ipsilateral fractures, open or pathological fractures, missing ultrasound scan information, and incomplete medical records.

A total of 214 cases of unstable HLCFs were retrospectively enrolled: 94 patients were excluded. Of these remaining 120 cases, 14 patients were failed UG-CRPP. Thus, a total of 106 patients (74 male patients and 32 female patients) were retrospectively included. The time from injury to surgery was 1–11 days. Overall, 55 cases were left-sided and 51 cases were right-sided.

This study was approved by the First Affiliated Hospital of the University of Science and Technology of China (approval number: USTC-FAH-2021-089), Fuyang People's Hospital (approval number: FYSRMLL-2021-38), and Ganzhou Maternal and Children's Health Care Hospital (approval number: FYBJY-2022-F05). All parents or their legal guardians provided informed consent on behalf of the patients to participate in the study.

### Surgical techniques

The same surgical techniques were used in all three hospitals by attending doctors. After general anesthesia, the patients were placed in a supine position, and the affected arm was placed on a C-arm platform. First, ultrasonography was used to confirm the Song type in multiple directions. For Song types 3–4, the affected arm was gently tracked into a straight position, and then varus was applied to create space for fragment reduction. The thumb of the surgeon compressed the fragment forward and upward, and then followed the valgus and flexed elbow. The reduction quality was assessed on coronal transverse, coronal anterolateral longitudinal, sagittal lateral longitudinal, and sagittal posterolateral longitudinal scans. For Song type 5, first, varus was applied to the affected arm, and the thumb was placed between the gap of the two fragments; second, valgus and flexion were applied to the elbow; third, the elbow alignment was checked by ultrasonography. If the reduction failed, a 2.0 mm k-wires was interposed into the fragment gap using prying to rotate and reduce the fragment. After the rotation deformity was addressed, the following techniques were used, as in types 3–4 (19). If there was a posteromedial dislocation of the elbow, the dislocated joint was first reduced, and then the procedure used for Song type 5 was followed. After successful reduction via ultrasound guidance, a 1.5-mm or 2.0-mm diameter k-wires was fixed along the transverse scan from the outermost cartilage of the lateral condyle to the medial epicondyle first, reducing the re-displacement of the distal cartilage hinge. The other two k-wires were then inserted into the metaphysis divergently. The elbow was then flexed to 90° and fixed with a long arm half cast in a neutral rotation position.

### Evaluation of peri-operation data and follow-up outcomes

Any unburied k-wires were removed after 4–6 weeks. Surgical, fluoroscopy times, and pinning times were recorded during the surgery. At the last follow-up, radiography was performed on the affected side to evaluate the Baumann angle, condylar shaft angle, carrying angle, lateral spur, and presence of capitellum necrosis. The normal Baumann angle, condylar shaft angle, and carrying angle were 71° ± 3.20°, 48.4° ± 3.40°, 11.0° ± 3.79°, respectively (20, 21). The lateral spur was evaluated using the intercondylar width ratio (22). Functionality was evaluated using the Mayo criteria (23). During follow-up, infection, nerve injury, cubitus varus/valgus, re-displacement, and nonunion were assessed.

### Statistical analysis

Statistical analyses were performed using IBM SPSS software (v.23.0; IBM Corp., Armonk, NY, USA). Data were presented as mean ± standard deviation. Continuous variables were analyzed using Student's *t*-test. Categorical variables were analyzed using the *χ*^2^ test or Fisher's exact test. Statistical significance was set at *p*-value <0.05.

## Results

### Peri-operation data

The incidence rate of UG-CRPP was 88%. The mean age of 106 patients was 5.0 ± 2.0 years, mean follow-up was 15.7 ± 4.4 months, mean surgical time was 54.6 ± 21.1 min, and mean x-way times was 9.3 ± 2.9 times. There was a significant difference (*p* = 0.01) between the flexion of the affected elbow (135.8° ± 6.9°) and that of the unaffected side (140.1° ± 5.9°). The extension of the affected side was 5.7° ± 4.9°, and that of the unaffected side was 5.8° ± 3.8°. The pronation of the affected side was 87.6° ± 2.3° and that of the unaffected side was 87.8° ± 2.6°. The affected side had a supination of 87.9° ± 3.1°, while the unaffected side had a supination of 88.0° ± 3.1°. There were no significant differences in terms of rotation and extension between the two sides (*p* > 0.05). The Mayo score of the affected elbow was 90.1 ± 4.8 points, with 38 children scoring over 95 points.

### Radiographic and clinical outcomes

**Figure 1:**
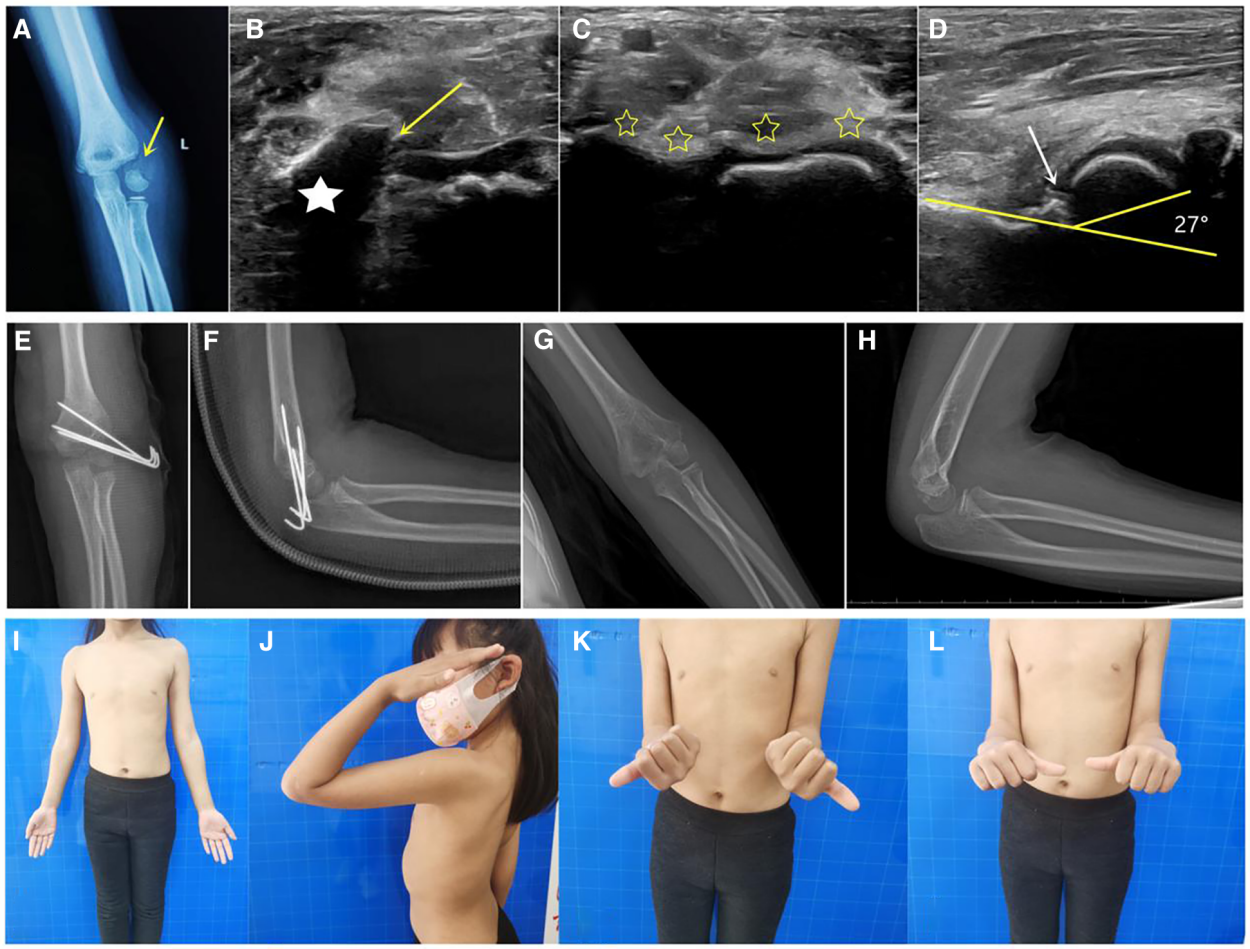
Case of a 6-year-old patient. (**A**) Radiograph showing the rotated and displaced left humeral lateral condylar fracture. (**B**) On ultrasonography, the humeral lateral condyle fracture was shown to have rotated, with the asterisk indicating the rotated fragment and the arrow indicating the fracture cartilage line. (**C**) Via ultrasound-guided closed reduction, the articular cartilage on the coronal plane was visible continuously (asterisk). (**D**) In the sagittal plane, the normal condylar shaft angle was 27°, without steps or displacement. (**E,F**) Immediate postoperative radiograph showing good fracture alignment. (**G,H**) Radiograph at the 19-month follow-up after surgery showing fracture healing and no capitellum necrosis. (**I–L**) At 19 months after surgery, the patient had good elbow function and no scarring.

At the last follow-up, the Baumann angle was 71.4° ± 5.4°, the condylar shaft angle was 39.9° ± 6.4°, and the carrying angle was 8.4° ± 3.6° on the affected elbow (Figure 1). According to the lateral spur measurement standard, there were 20 normal cases, 70 minor spurs, and 16 moderate spurs.

### Complications

Fourteen patients developed superficial pin infections, no deep osteomyelitis, or arthritis. One patient experienced mild postoperative displacement. None of the patients had bone bar, varus, or valgus deformities. One patient had capitellum necrosis, and the ossified capitellum was divided into two parts. No evidence of delayed union, nonunion, or nerve injury was observed.

### Criteria for UG-CRPP

**Figure 2:**
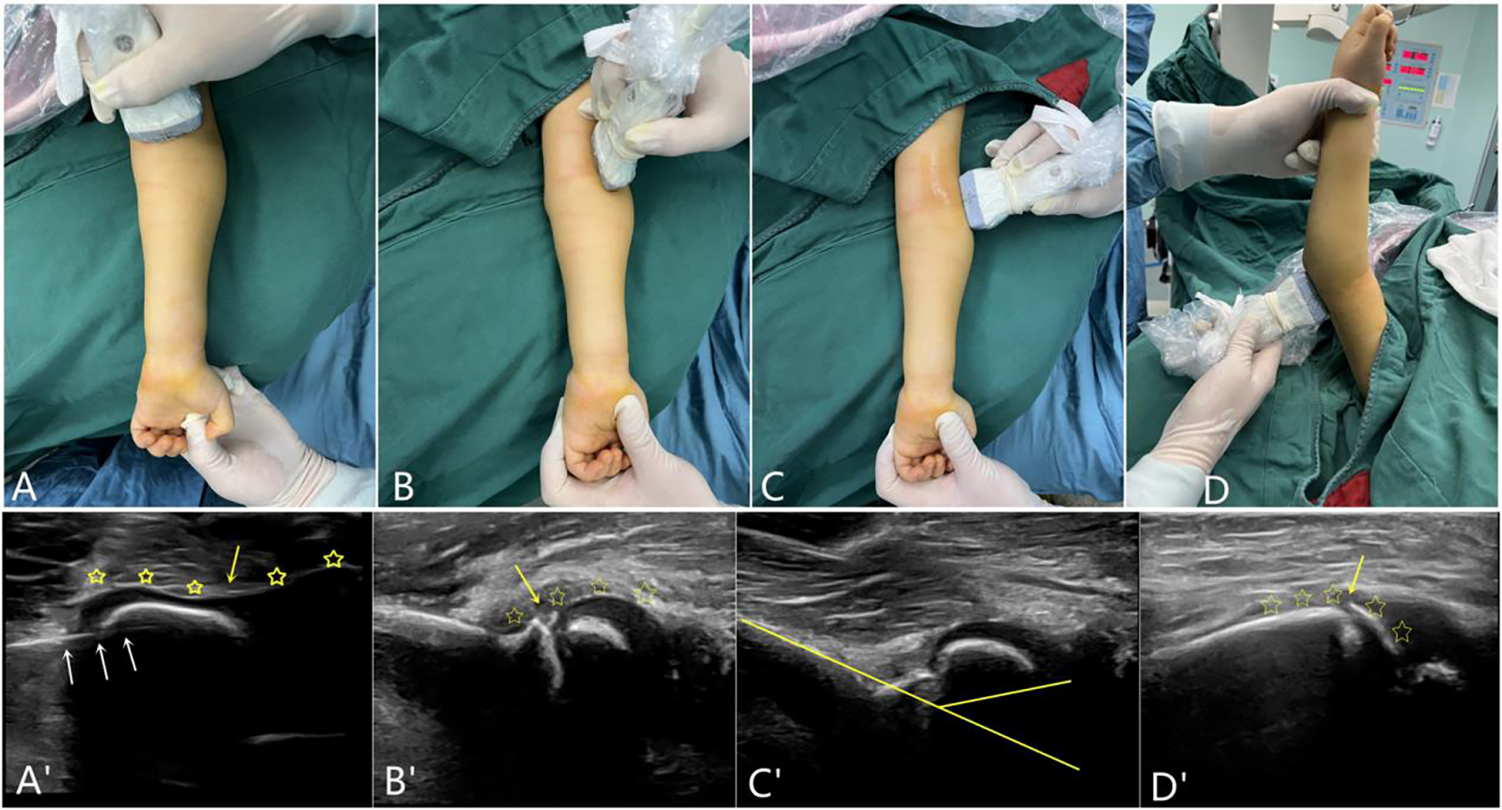
The probe position and corresponding scans on ultrasonography. (**A**) is the scanning probe position of the front coronal plane parallel to the articular surface. (**A**)’ shows no anteroposterior or lateral steps (yellow stars) or fracture line (yellow arrow) in the cartilage, and the white arrows indicate the k-wires. (**B**) indicates the scanning of the vertical articular surface of the anterolateral coronal plane. (**B**)’ shows no displacement of the fracture; the distal end of the condylar and the capitellum are continuous. (**C**) indicates the lateral sagittal plane vertical articular surface scan, (**C**)’ shows no displacement of the fracture; the condylar shaft angle was normal. (**D**) indicates the vertical articular surface scan of the posterolateral sagittal plane; the fracture line shown in (**D**)’ is essentially continuous.

The success criteria were assessed mainly in four scans of patients. The probe positions and ultrasonographic images are indicated as X and the corresponding X’ in Figure 2 and are defined as follows:

A. Coronal plane parallel articular surface scanning: absent or less than a cartilage thickness step on the cartilage hinge.
B. Coronal plane vertical articular surface scanning: no lateral displacement; the distal end of the condyle and capitellum is intact.
C. Sagittal plane vertical articular surface scanning: no anteroposterior displacement and less than a cartilage thickness step.
D. Posterolateral sagittal plane vertical articular surface scanning: the posterior fracture line is intact or less than a cortical step.

The intra- and interobserver agreement for successful criteria for UG-CRPP across all observers was substantial with κ values of 0.617 (95% CI: 0.481–0.783) and 0.563 (95% CI: 0.412–0.865), respectively.

## Discussion

According to Song's criteria, unstable HLCFs are traditionally treated by open reduction with k-wires fixation to ensure anatomical reduction of the cartilage hinge. In recent years, UG-CRPP has became popular and achieved good results in children (24–26). Ultrasonography can reveal the displacement, shape, rotation, alignment, and condylar shaft angle of the cartilage hinge. During the HLCFs reduction process, the alignment of two fragments can be clearly observed, and the live first intra-cartilage k-wires fixation process reduces the pinning penetration time (13, 18, 24–27).

Compared with previous open reduction methods, the UG-CRPP surgical method has the advantages of no radiation, amenability to multidimensional assessment of reduction quality, minimal invasive, and circumvention of the need to strip the soft tissue of the humeral epicondyle to expose the joint surface. This novel technique protects blood circulation, reduces k-wires penetration time, minimizes cosmetic concerns, and yield high patient satisfaction.

Many studies have evaluated arthrography to assist CRPP. However, arthrography only shows a single coronal plane of the articular cartilage and provides limited help in the sagittal plane (5–8). Compared to CRPP with arthrography, UG-CRPP has a higher success rate, is more precise, and requires less surgical time (8, 9). Under the guidance of arthrography, 75% of patients achieved a closed reduction in the study by Song et al. (5). In another study, half of the reductions were successful in patients with Song type 5, 75% in those with Song type 4, and 76% in those with Song type 3 fractures (28). The UG-CRPP success rate was 88% in this study, which is higher than that obtained in the above reports, indicating that it is easier to achieve CRPP with ultrasound guidance. In addition, the advantages of surgical time, fluoroscopy frequency, and aesthetic problems compared to arthrography-assisted CRPP are significant (28). Furthermore, ultrasonography can clear the cartilage hinge fracture line and reduce k-wires penetration times with direct vision, which may avoid physis damage and lead to fewer complications.

For unstable HLCFs, many studies have evaluated UG-CRPP with open reduction, confirming the feasibility and effectiveness of UG-CRPP (17, 28–30). Indeed, although open reduction can effectively remove blood scabs or other fibers within the joint, there is no significant difference in the overall incidence of complications between open and closed reduction (28). Compared with the previous studies, this study was a three-center study with a larger sample size and provided evidence of good results, further confirming the effectiveness of UG-CRPP.

Although UG-CRPP techniques have become popular, there are currently no definite probe positions or corresponding imaging criteria indicative of successful outcomes (13–19). This study describes the probe position of UG-CRPP in four scans: (i) coronal plane parallel articular surface scanning to evaluate the lateral and anteroposterior displacement of the cartilage hinge, (ii) coronal plane vertical articular surface scanning to evaluate the anteroposterior displacement of the fragment and capitellum-radial alignment, (iii) sagittal plane vertical articular surface to evaluate the condylar-shaft angle, and (iv) sagittal plane posterolateral scanning to evaluate the metaphyseal fracture gap. Four scans were performed to assess the quality of the fracture reduction. If the first and second scans were successful, closed reduction could be usually achieved. In practice, ultrasonographic evaluation achieves a local amplification effect, which indicates that a minimal fracture gap is clearly visible on ultrasonography but is difficult to display on x-ray. Nonetheless, the evaluation criteria for HLCFs require further large-scale multicenter studies to confirm their clinical feasibility.

In terms of complications, compared with other UG-CRPP studies, no increase in either the surgical time or the incidence of postoperative complications was found in this study (13–19). One patient, a young toddler possibly presenting with a less stable fixation, exhibited mild re-displacement after surgery. In another patient, the ossification of the capitellum was divided into two parts, and it was necessary to continue the follow-up and observe the development of the capitellum. In this study, 16 patients developed moderate lateral spurs. Compared with previous open reduction, the formation of a lateral spur was observed less frequently, which may be related to non-stripping of the lateral periosteum and protection of the blood supply, thereby reducing excessive proliferation. Most complications occurred in the first 3 months of this technique, which indicated that after a certain learning curve, surgical outcomes improved.

This study had several limitations. First, it was a retrospective study with surgical interventions conducted by three different surgeons; thus, we cannot eliminate potential bias in outcomes due to differences in surgical skill levels. Second, this study did not include fracture subtypes and could not address the relationship between the fracture subtypes and outcomes. Third, the success criteria for UG-CRPP should be confirmed in a multi-center prospective control study. Finally, this study only compared contralateral function and radiographs, thus reducing the effectiveness and feasibility of UG-CRPP.

## Conclusion

UG-CRPP is a reliable surgical option for the treatment of unstable HLCFs with minimal blood loss, less invasive, no radiation, better cosmetic outcomes, and fewer complications. Four levels of evaluation for ultrasonography criteria were defined to establish successful outcomes.

## Data Availability

The raw data supporting the conclusions of this article will be made available by the authors, without undue reservation.
